# Correlation between the risk of lymph node metastasis and the expression of GBP1 in breast cancer patients

**DOI:** 10.12669/pjms.40.1.8251

**Published:** 2024

**Authors:** Yukun Liu, Ziying Wu, Jun Lin, Zhimei Wang

**Affiliations:** 1 Yukun Liu Department of Breast Surgery, Breast Disease Center, The Affiliated Qingdao Central Hospital of Qingdao University, Qingdao 266042, Shandong Province, P.R. China; 2 Ziying Wu Department of Colorectal and Anal Surgery, The Affiliated Qingdao Central Hospital of Qingdao University, Qingdao 266042, Shandong Province, P.R. China; 3 Jun Lin Department of Breast Surgery, Breast Disease Center, The Affiliated Qingdao Central Hospital of Qingdao University, Qingdao 266042, Shandong Province, P.R. China; 4 Zhimei Wang Department of Gynecological Neoplasms, The Affiliated Qingdao Central Hospital of Qingdao University, Qingdao 266042, Shandong Province, P.R. China

**Keywords:** Breast cancer, Lymph node metastasis, Guanylate-binding Protein-1, Risk factors

## Abstract

**Objective:**

To explore the prognostic value and correlation between the risk of lymph node metastasis (LNM) and Guanylate-binding Protein 1 (GBP1) in breast cancer (BC) patients.

**Methods:**

In this retrospective study, the clinical data of 150 patients with BC who were surgically resected in The Affiliated Qingdao Central Hospital of Qingdao University from January 2019 to December 2021 were included. Patients were divided into metastasis group (n=110) or non-metastasis group (n=40) according to whether there was LNM post-surgery. Logistic regression was used to analyze the risk factors for LNM in BC, and Kaplan-Meier was used to assess the risk of disease progression 12 months post-operation in both groups. Patients were divided into a GBP1 low expression-group (n=75) or a GBP1 high expression-group (n=75). The risk of disease progression, one-year post-surgery was analyzed, and the predictive value of GBP1 in BC tissue was assessed by the receiver operating characteristics (ROC) curve.

**Results:**

Independent risk factors for BC with LNM were GBP1, CEA and TNM stage (P<0.05). There is a linear relationship between GBP1 expression and LNM risk in BC (*χ^2^*=0.88, P<0.05). Patients with high expression of GBP1 had a higher risk of LNM (*χ^2^*=3.204, P<0.001) and early postoperative progression (*χ^2^*=7.412, P<0.05). The AUC of GBP1 in predicting the risk of LNM was 0.840.

**Conclusions:**

Patients with BC and a higher expression of GBP1 could be at an increased risk of LNM. Elevations in GBP1 expression can also suggest a poor prognosis for patients with BC.

## INTRODUCTION

Breast cancer (BC) is the most common form of malignancy among women in the world.1 In 2020, there were 2.3 million new cases of BC and 685 000 deaths globally.^2^ Typically, symptoms such as a lump on the breast, nipple discharge, and abnormal breast skin and areola are present.^3^ Lymph node metastasis (LNM), which is a primary cause of increased mortality in BC, can occur if timely and effective treatment is not taken.^1^,^3^

Currently, axillary lymph node dissection (ALND) and chemotherapy are widely used in the clinical diagnosis and treatment of BC with LNM.^4^,^5^ However, years of practice have shown that ALND causes minimal surgical trauma complications such as lymphedema and decreased arm mobility.^3^,^4^,^6^ Additionally, nausea and vomiting, alopecia, liver and kidney damage caused by chemotherapy are side effects which can seriously affect patient prognosis.^7^ Therefore, it is important to evaluate the risk of LNM and identify the relevant influencing factors of BC with LNM to improve the prognosis and quality of life of patients.

Previous research has identified GBP1 as a monomer initiating protein which acts as a carcinogen in the occurrence, development, invasion and metastasis of BC.^8^ Additionally, the risk of brain metastasis is increased through T lymphocyte induction of GBP1 overexpression within BC cells.^9^ However, the correlation between GBP1 and the risk of LNM in BC patients and the predictive value of GBP1 on prognosis still need further analysis.^8^,^9^

This study was planned because there is limited research examining the correlation between the risk of BC in patients with LNM and the expression level of GBP1. Therefore, this study analyzed the clinical data of 150 patients with BC treated in our hospital to explore the correlation between the risk of LNM in BC patients and the expression level of GBP1.

## METHODS

In this retrospective study, one hundred and fifty patients with BC who were admitted in The Affiliated Qingdao Central Hospital of Qingdao University for surgical resection from January 2019 to December 2021 were included. Based on the clinical data of the patients, they were divided into metastasis group (n=110) or non-metastasis group (n=40) according to whether there was LNM post-surgery. The pathological diagnosis of BC was confirmed by two pathologists with more than five years of experience in our hospital.

### Inclusion criteria:

- Patients in generally good condition, without serious disease, and can tolerate routine examination and treatment.
- Patients diagnosed with BC by pathology (WHO standard) after puncture of suspicious masses and those with benign lesions that need surgical treatment after assessment.^10^
- Patients with no lymph node metastasis confirmed by intraoperative pathological examination.
- Patients who had not received radiotherapy and chemotherapy before operation.
- Patients with complete clinical data.

### Exclusion criteria:

- Patients with severe disease of important organs such as heart, liver, lung, and kidney.
- Bilateral BC.
- Patients with other primary malignant tumors.

### Ethical Approval

This study has been approved by the Medical Ethics Committee of our hospital (approval number: KY202107202; date: 2021-11-23)

Baseline clinical data of patients was collected, including age, lactation history, family history of BC, BMI, TNM stage, carcinoembryonic antigen (CEA), menstruation, and molecular typing. All patients were followed up for 12 months. If the patient developed recurrence, distant metastasis, or died post-surgery, it was defined as disease progression. According to the median level of GBP1 (1.80), patients were divided into the GBP1 low expression group (75 cases) or the GBP1 high expression group (75 cases).

### Detection of the expression level of GBP1

The samples (3-5m^3^) were collected during the operation and were immediately frozen in liquid nitrogen and transferred to -80^0^C for storage.

### Immunohistochemical detection

The BC tissue was embedded in paraffin, sectioned (4μm) and placed on slides. The slides were dewaxed, and antigen retrieval was performed. Serum blocking was performed, and the slides were stained with primary anti-GBP1 and secondary anti-GBP1 and given a streptavidin biotin complex (SABC) treatment and chromogenic agent treatment. The slides were re-stained, dehydrated, and blocked again.

### Western blotting protein electrophoresis

Protein samples for GBP1 were extracted and equal amounts of protein were run through polyacrylamide gel electrophoresis (SDS-PAGE) and transferred onto a PVDF membrane. Membranes were blocked and detection of GBP1 antibodies was performed. Protein was quantified using ECL chemiluminescence and data was collected and analyzed with image processing system (Image-J software, National Institute of Health (NIH), Bethesda, USA).

### Fluorescence quantitative PCR detection

Total RNA was extracted, and reverse transcription was performed. PCR was quantified results were analyzed and processed.

### Statistical analysis

SPSS 26.0 (IBM, USA), R V4.0.5 (RStudio Inc., USA) and GraphPadPrism8.0 software (Dotmatics, USA) were used for data analysis. The measurement data were expressed as mean ± standard deviation (*χ̅*±*S*). The count data were expressed in percentage (%), and the comparison between groups was performed by *χ^2^* test. The rank sum test was used for grade data and multivariate logistic regression model was used to analyze the risk factors of LNM in BC patients. R language, RMS and ggplot2 software package were used to draw a restricted cubic bar graph to analyze the correlation between different levels of expression of GBP1 and the disease progression of patient’s post-surgery. The Kaplan - Meier survival analysis was used to analyze the risk of disease progression 12 months post-operation in the two groups. The ROC was drawn and the area under the curve (AUC) was calculated to test the value of the expression level of GBP1 in predicting the risk of LNM in BC patients. Statistical significance was set at *P* <0. 05.

## RESULTS

The average age of the metastasis group was 59.26±8.27 years, and their average body mass index (BMI) was 22.71±2.42kg/m^2^. The average age of the non-metastasis group was 58.07±6.77 years, and their average BMI was 23.18±2.24 kg/m^2^.

The postoperative LNM rate of patients with BC was 26.7% (40/150). BC with LNM was considered the dependent variable, while age, lactation history, family history of BC, BMI, TNM stage, CEA, menstruation, and molecular typing, were independent variables (Table-I). The univariate logistic analysis showed that elevated GBP1 and CEA expression, TNM stage and molecular typing were risk factors for BC with LNM, with a statistically significant difference (P<0.05; Table-II).

**Table-I:** Assignment of independent variables.

Variables	Assignment method
GBP1 level	Low=0, High=1
TNM stages	T1N0M0=0, T1N2M1=1, T2N3M1=2
CEA	Low=0, High=1
Molecular typing	Luminal A=0, Luminal B1=1, Luminal B2=2, TNBC=3

**Table-II:** Univariate logistic analysis of BC with LNM.

Factors	Metastasis-group(n=110)	Non-metastasis-group(n=40)	*t/χ^2^*	*P*
Age (years)	59.26±8.27	58.07±6.77	-0.814	0.417
Breastfeeding history (yes)	97 (88.2)	36 (90.0)	0.096	0.756
BMI (kg/m^2^)	22.71±2.42	23.18±2.24	1.803	0.28
Family history of BC (Yes)	59 (53.6)	16 (40.0)	2.182	0.14
GBP1 level	1.51±0.51	1.81±0.39	-3.395	0.002
***TNM stages***				
T1N0M0	12 (10.9)	19 (47.5)	25.755	<0.001
T1N2M1	47 (42.7)	14 (35.0)		
T2N3M1	51 (46.4)	7 (17.5)		
CEA (ng/mL)	20.86±2.49	18.68±1.70	-6.103	<0.001
Menstruation (menopause)	61 (55.5)	25 (62.5)	0.595	0.44
***Molecular typing***				
Luminal A	17 (15.5)	15 (37.5)	8.572	0.036
Luminal B1	48 (43.6)	13 (32.5)		
Luminal B2	32 (29.1)	8 (7.3)		
TNBC	13 (11.8)	4 (10.0)		

**Fig.1:**
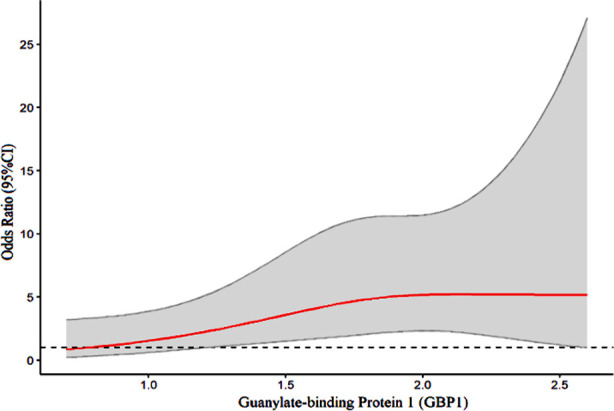
Correlation between the expression level of GBP1 and the risk of lymph node metastasis (LNM) in patients.

The multivariate logistic analysis showed that high levels of GBP1 and CEA expression and TNM stage were independent risk factors of BC complicated with LNM (P<0.05; Table-III). There is a linear relationship between the level of GBP1 and the risk of LNM in BC patients, χ2=0.88, P>0.05; Fig.1).

**Table-III:** Multivariate Logistic Regression Analysis of BC with LNM.

Factors	β	S·E	Wald	P	OR	95%CI
High level of GBP1	1.115	0.499	4.985	0.026	3.049	1.146-8.113
High level of CEA	0.461	0.139	11.004	0.001	1.585	1.207-2.081
T1N0M0		7.629		0.022		
T1N2M1	1.381	0.604	5.222	0.022	3.979	1.217-13.006
T2N3M1	1.926	0.732	6.919	0.009	6.862	1.634-28.820

**Fig.2:**
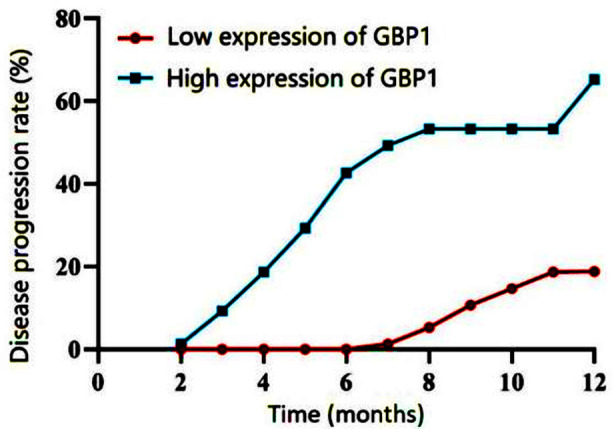
Survival curve of patients with different levels of GBP1. The survival analysis curve was drawn with a median GBP1 expression of 1.8 in 150 patients with BC.

**Fig.3:**
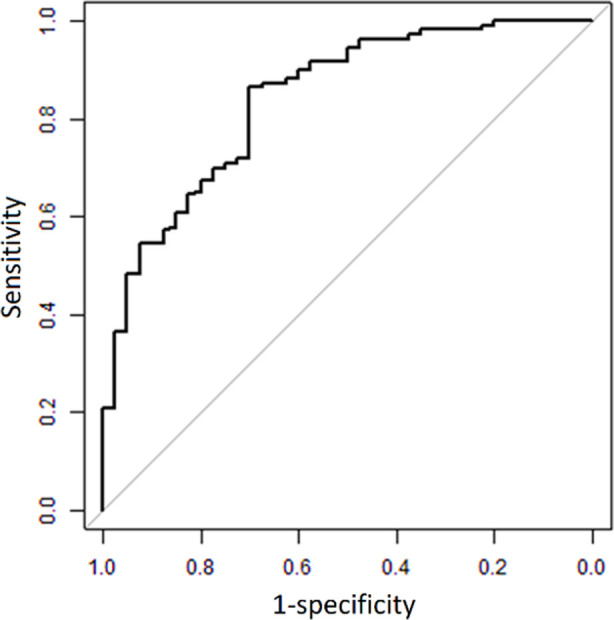
ROC curve of lymph node metastasis (LNM) risk value predicted by GBP1 in patients with breast cancer.

The survival analysis, median GBP1 expression of 1.8 in 150 patients with BC, showed that elevations in GBP1 expression results in shorter survival compared to low-level expression of GBP1 (*χ^2^*=3.204, P<0.001; Fig.2). The area under the curve (AUC) of the receiver operating characteristic curve (ROC) pertaining to the level of GBP1 needed to predict the risk of LNM is 0.840, suggesting good predictive value (Fig.3).

## DISCUSSION

The results of this study showed that high expression of GBP1 and CEA and TNM stages were independent risk factors for BC with LNM. These results are different to previous work by Grant et al^11^ who found that older age and family history of disease in female BC patients were related to tumor occurrence and complications.^12^ Most of the patients included in this study were young to middle-aged, and may have healthy hormone secretion and more effective immune system regulation.^13^ Further, pregnancy status, lactation history, menstrual cycle disturbances, and menopause all affect hormone concentrations, thereby increasing the risk of BC, but the association between these factors, BC and LNM has not been established.^12^ The placenta and pregnancy can modulate the maternal immune system, but such effects will gradually recover after delivery.^12^,^14^,^15^

Patients with multiple tumor types often have lower BMI, suggesting that a decrease in BMI may be a marker for tumor progression and metastasis.^16^5 days a week for 5-8 weeks. To understand the characteristics of radioresistant cancer cells and to develop more effective radiotherapy, we established a series of novel, clinically relevant radioresistant (CRR However, our results did not find BMI to be a risk factor for LNM of BC, which is inconsistent with the findings by Wang et al.^17^ As a monomer starting protein, GBP1 can combine with GTP, GDP and GMP.^18^ Some studies have shown that the increased expression of GBP1 in the inflammatory reaction period is related to the activation of inflammatory factors such as INF-γ, which can resist the invasion of inflammation by inhibiting the proliferation of epithelial cells.^19^ Yamakita et al.^20^ also found that GBP1 has a significant role in promoting the occurrence of lung adenocarcinoma and improving tumor invasion through immunohistochemistry and case analysis and control.

The level of GBP1 expression also plays an important role in the process of LNM in patients with BC, which was also confirmed by our results.^19^-^21^ High GBP1 expression is an independent risk factor for BC with LNM, indicating that GBP1 acts as a carcinogen in the development, invasion and metastasis of BC.^22^ Zhu et al.^23^ found that GBP1 was significantly overexpressed in the molecular typing of BC triple negative breast cancer (TNBC) and when GBP1 expression was reduced, the TNBC cell lines stopped growing. In comparison with non-TNBC tissues, the overexpression of GBP1 in the TNBC group correlated with a poor prognosis in TNBC patients. These results suggest that molecular typing of BC is associated with high expression of GBP1, LNM and poor prognosis.^23^,^24^

TNM staging is an established BC risk assessment index system which can predict the recurrence and metastasis of BC.^25^,^26^ TNM staging represents the size and location of the tumor, the lymph nodes where cancer cells have spread, and the degree of metastasis. In this study, TNM system staging of BC was an independent risk factor for LNM, which further explains the accuracy and sensitivity of TNM staging for BC and LNM. In addition, CEA is a tumor-related antigen extracted from embryonic tissue which generally exists in human endoderm cells. When the cell tissue becomes cancerous, CEA differentiates from endoderm cells into cell membrane structural proteins, forms in the cytoplasm, and is secreted into the body through the cell membrane.^27^,^28^ CEA expression is relatively stable and represents the normal operation of blood vessels and can also be used as an indicator of endocrine function.^29^ However, when breast cell disease occurs, CEA will increase abnormally, and overexpression will also induce the production of excessive protein tyrosine kinase, suggesting CEA expression level is closely related to the process of LNM in BC.

### Limitations

It is a retrospective study with a relatively small samples, the generalization of the findings is limited. Future prospective studies should be conducted to verify the findings. Furthermore, only a few risk factors were investigated in this study, other risk factors such as history of drinking or smoking should be studied.

## CONCLUSION

Elevations in GBP1 and CEA expression and TNM staging are independent risk factors for BC with LNM. BC with LNM is not necessarily related to patient age, lactation history, family history of BC, BMI, menstruation, or molecular typing, Patients with high GBP1 expression have a relatively short overall survival period, and suggests poor patient prognosis.

### Authors’ contributions:

**YL:** Conceived and designed the study.

**ZW**, **JL** and **ZW:** Collected the data and performed the analysis.

**YL:** Was involved in the writing of the manuscript and is responsible for the integrity of the study.

All authors have read and approved the final manuscript.
